# Differences in Astringency Subqualities Evaluated by Consumers and Trained Assessors on Sangiovese Wine Using Check-All-That-Apply (CATA)

**DOI:** 10.3390/foods10020218

**Published:** 2021-01-21

**Authors:** Alessandra Rinaldi, Riccardo Vecchio, Luigi Moio

**Affiliations:** 1 Biolaffort, 126 Quai de la Souys, 33100 Bordeaux, France; 2 Department of Agricultural Sciences, Division of Vine and Wine Sciences, University of Naples Federico II, Viale Italia, angolo via Perrottelli, 83100 Avellino, Italy; riccardo.vecchio@unina.it (R.V.); moio@unina.it (L.M.)

**Keywords:** astringency, subquality, CATA, trained assessors, consumers, liking, Sangiovese

## Abstract

The astringency of red wine represents an important factor of quality and liking evaluation by consumers, but it is sometimes associated to a negative feature. We studied the differences in astringency subqualities of Sangiovese wines between consumers and trained assessors. Wines belonging to three denominations (Chianti Classico, Toscana, Morellino di Scansano) and a Chianti Classico specification (Chianti Riserva), from three price ranges (low, medium, high) were evaluated. Regular wine consumers and trained panel assessed the wines applying the Check-All-That-Apply (CATA) questionnaire relative to six astringency attributes (silk, velvet, dry, aggressive, hard, mouthcoat). Differences between panels were more associated with the high-price wines, which were characterised by negative subqualities for consumers. Preference maps revealed that mouthcoat was the term mainly associated with consumers’ liking, while other subqualities as persistent, rich, and full-body, provided by the trained assessors, may represent the drivers of liking for Sangiovese wine. This study has demonstrated that a trained sensory panel provides highly valuable information regarding the mouthfeel characteristics of Sangiovese wines and the attributes driving consumer liking.

## 1. Introduction

Wine is a multisensory product, and its quality is defined by intrinsic and extrinsic cues [1,2]. Sensory characteristics, such as color, odor, aroma, taste and mouthfeel, are considered intrinsic wine qualities. Extrinsic qualities, such as brand name, region and country of origin, label, presence/absence of awards, have been shown to affect the way intrinsic cues are evaluated and can affect the overall perception of wine quality [3,4]. Depending on involvement and cultural level, consumers are highly influenced by extrinsic qualities when evaluating wine, although the intrinsic tasting experience is the most important reason for drinking wine [5]. Wine experts or trained assessors, on the contrary, can focus their attention on specific sensory inputs and analyse wine according to their improved chemosensory acuity [6].

Analysing hedonic liking and perceived quality of Californian Cabernet Sauvignon wines, Hopfer and Hyemann [7] revealed that a consistent segment of consumers preferred low-quality wines. In addition, consumers’ liking patterns were drastically different from experts’ quality perceptions. Similarly, applying descriptive analysis, Lattey and colleagues [8] showed that consumer groups had contrasting preferences from experts, which considered wines with higher hotness, astringency, and fruit and oak flavor as of superior quality. It is probable that besides the level of expertise, training, interest, and knowledge of subjects, the choice of the sensory method may play an important role in wine evaluation by experts and novels.

Recently, Check-All-That-Apply (CATA) questions have been introduced as rapid approaches for sensory analysis [9], presenting a pre-defined list of sensory descriptors to participants, which are instructed to select all that apply. This method is considered by the consumer to be easy and not tedious to complete [10,11], and generates similar responses between trained assessors and consumers in product characterization [12,13]. Regarding wines, this approach has been applied to evaluate the sensory profile of wine [14], and to identify the terms used by consumers to describe red wine astringency [15]. More recently, a combination of CATA questionnaire and training with touch-standards was used to evaluate the qualitative aspects of red wine astringency under different conditions [16,17,18].

The astringency of red wine is an important parameter for quality and liking judgment but is sometimes perceived as a negative feature and therefore rejected by consumers. However, the hedonic responses vary among consumers and wine styles. Some consumers like the wine more as astringency increases, whereas others have a negative response to this intensification [19]. In a high-tannic wine such as Tannat, astringency was positively correlated to quality, influencing the flavor (attack, evolution and persistence), body, and overall quality of the wine [20]. In this scenario, as red wine can show different qualitative characteristics, both positive and negative [21], astringency may have a different impact on consumers’ appreciation. To the best of our knowledge, no studies are dealing specifically with the differences in astringency subqualities between consumers and trained assessors.

The current research was carried out on the Sangiovese, one of the most renown and consumed wines in Italy and abroad. The astringency subqualities of Sangiovese wines belonging to three denominations (Chianti Classico, Toscana, Morellino di Scansano) and a Chianti Classico specification (Chianti Riserva), from three price ranges (low, medium, high) were evaluated. Two panels, one formed of 150 regular wine consumers and one of trained assessors, evaluated the mouthfeel characteristics of Sangiovese wines using the CATA questionnaire relative to six attributes (silk, velvet, dry, aggressive, hard, mouthcoat). CATA profiles were compared by Multiple Factor Analysis (MFA). To assess the effect of astringency sensations on the liking of Sangiovese wines by consumers, two references maps (with data from consumers and trained assessors) were also produced.

## 2. Materials and Methods

### 2.1. Wine Samples

Twelve Sangiovese wine samples belonging to three denominations (Chianti Classico DOCG—Denominazione di Origine Controllata e Garantita-CC, Toscana IGT-Indicazione Geografica Tipica-TS, Morellino di Scansano DOCG-MS) and a Chianti Classico DOCG specification (Chianti Riserva DOCG-CR), from three price ranges (low: CCL, TSL, MSL, CRL; medium: CCM, TSM, MSM, CRM; high: CCH, TSH, MSH, CRH) were selected and bought from the largest national, online retail store. The wines belonged to the same 2015 vintage. The price ranges represented the lower quartile, the median, and the upper quartile of the distribution of retail prices per bottle (0.75 cL) in Italian online wine stores at the time of the research; specifically, the basic wine was priced € 5 (L), the medium € 10 (M), and the high € 20 (H).

### 2.2. Wine Evaluation by Trained Assessors

A trained panel composed of five females (aged 35–50 years) and eight males (25–44) participated in the wine evaluation sessions. The thirteen assessors were previously trained for the evaluation of astringency and mouthfeel sensations, as described in Rinaldi and Moio [16]. Briefly, panellists were first introduced to the theory of astringency. They were then familiarised with astringency rating by tasting water solutions and white wine spiked with five different enological tannins (from 0.1 g/L to 5.0 g/L in water and from 0.1 g/L to 1.5 g/L in white wine) on a 9-point scale (named: absent, very weak, weak, weak moderate, moderate, moderately strong, strong, very strong, extremely strong). A discussion on the perception of subqualities according to the mouthfeel wheel was carried out after tasting [21,22]. The most familiarised terms were selected among the 33 astringency definitions. Only terms cited by more than 20% of the judges on the panel were considered [23] and were introduced in the Check-All-That-Apply (CATA) questionnaire [24]. CATA questions are a form of multiple-choice question where a list of 16 subquality terms (sensations of touch, taste, and flavor) are presented and subjects tick the options that they consider to be applicable to the wine. In order to further deepen the insight into the subqualities of astringency, the CATA method was coupled with the use of touch standards as described by different authors [25,26]. The novelty consisted in placing the standards in a closed box to avoid visual bias and to enhance the tactile sensation felt by mechanoreceptors in the fingers [27,28]. Training for astringency subqualities was done by evaluating six commercial red wines spiked with 0.2 g/L to 0.5 g/L of five enological tannins, using CATA questions and descriptors as described in [16]. Sangiovese wines were evaluated twice. In each session, two tasting evaluations of four anonymous samples were performed. They were presented in balanced, random order at room temperature (18 ± 2 °C) in black tulip-shaped glasses coded with 3-digit random numbers. The assessors were instructed to pour the whole sample in their mouth, hold it for 8 s, expectorate and evaluate it using the CATA questionnaire with the six subqualities (three negative: dry, aggressive, hard; and three positive: silk, velvet, mouthcoat), checking the subqualities if present. Attributes were presented in balanced order between and within panelists following a Williams Latin square design [29]. Trained assessors waited for 30 s, then drank mineral water (Sorgesana, pH ≈ 7), and waited for 30 s again. Then, assessors took another sip and rated the intensity of the subquality sensation (Rate-All-That-Apply, RATA) using sixteen attributes (silk, velvet, dry, corduroy, adhesive, aggressive, hard, soft, mouthcoat, rich, full-body, green, grainy, satin, pucker, persistent) defined in Table 1.

The applied scale for RATA was 0–5 points with end-point anchors 1 = ‘slightly applicable’ and 5 = ‘very applicable’. Judges waited for 2 min before rinsing with mineral water for 10 s twice, and then waited at least 30 s before the next sample. Samples were tasted more than once.

### 2.3. Wine Evaluation by Consumers

Participants were recruited by an external consumer association, applying three specific screening criteria: aged above 21; buying a bottle of wine at least once a month, and consuming wine at least once a week. 150 regular wine consumers evaluated the Sangiovese wines. Participants expressed their general liking for Sangiovese wines (*a priori* liking *M* = 4.4). Individual wine involvement was measured (*M* = 5.1) together with wine knowledge (*M* = 4) (for a detailed overview of the dataset see [30]). The final sample was composed of 62% male, 42% completed secondary school, and 36% graduated from university; the average age was 41.6 years old (*S.D*. 13.7), and the range age was between 21 and 75 years old; 57% of subjects consumed wine three or four times a week; 26% paid, on average, a bottle of wine for home consumption between 9 € and 15 €.

Experimental sessions were organised randomly assigning 15 ± 3 participants to each session, applying a between-subject design. A total of eleven experimental sessions were performed over five weekdays and lasted around one hour. Between 12 and 18 subjects participated in each session. In each session, all respondents tasted three wines—opened 1 h before the beginning of the session and served at 18 ± 2 °C in ISO glasses—and strictly received information on the wine category (denomination of origin and specification) through observation of the bottle labelling. Subjects were aware of the correspondence among wines and bottles. Samples, presented one by one, were randomized across sessions according to a Williams’ Latin square design, balanced for order and first-order carry-over effects. Before starting tasting sessions, consumers were provided with definitions of the six attributes of astringency (silk, velvet, dry, aggressive, hard, mouthcoat) presented in the CATA questionnaire, as described in Table 1. Attributes were presented in balanced order between and within participants following a Williams Latin square design. In order to minimize dumping, an option for ‘Other’ was included with an open-ended text box; these data were not used in any analysis. Before CATA evaluation, consumers expressed their liking according to a 9-point hedonic scale (1 = ‘dislike extremely’, 9 = ‘like extremely’). Consumers were encouraged to wash their mouth between each tasting with still mineral water and unsalted crackers.

The protocol used for data collection complied with national ethical requirements and was carried out in accordance with The Code of Ethics of the World Medical Association (Declaration of Helsinki) for experiments involving humans. In particular, all subjects provided their informed consent to participate in the study, and all data were collected anonymously. Data were also recorded and managed according to the Italian Personal Data Protection Code (Law Decree no. 196 of 30 June 2003).

### 2.4. Data Analysis

Cochran’s Q test, which was followed by the multiple pairwise comparison test (*p* < 0.05), was applied to the CATA data to identify significant differences in the frequency of use of the terms (Cf%) between consumers and trained assessors for each of the terms included on the CATA questionnaire. Analyses were carried out by the XLSTAT software package (Addinsoft, XLSTAT 2017). MFA was used to assess the configurational similarity of wine spaces obtained with CATA data by the consumer and trained panels. Principal Component Analysis (PCA) and MFA were used to model liking scores from consumers as a supplementary variable RATA data (from trained assessors) and with CATA data (for consumers), respectively. MFA was performed using FactoMineR [31] package.

## 3. Results

### 3.1. CATA Comparison between Trained Assessors and Consumers

**Figure 1:**
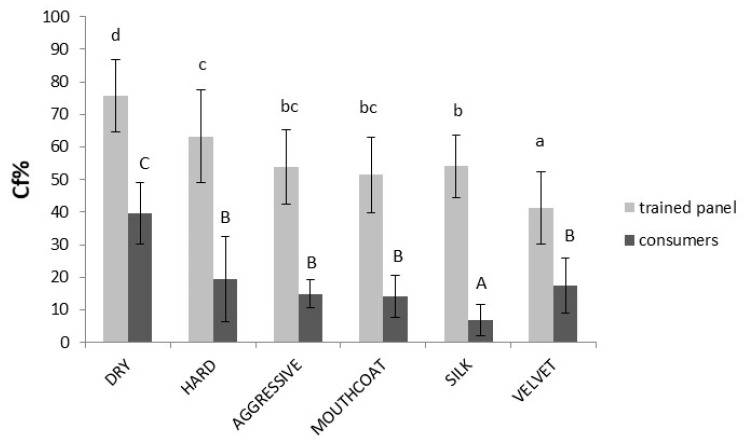
The average citation frequencies (Cf%) of the astringency subqualities in all Sangiovese wines (*n* = 12) by trained panel and consumers. The upper letters indicate statistical differences in subqualities between the consumers and the lower letters between the trained panel (*p* < 0.05).

The terms mainly used by the trained panel and consumers to describe the Sangiovese wine are shown in Figure 1. The citation frequencies (Cf%) are the average of all wines (*n* = 12) for each subquality.

**Figure 2:**
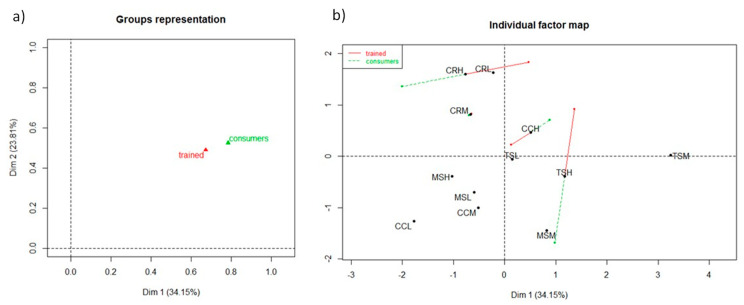
Differences in evaluating the astringency subqualities by CATA between trained assessors and consumers of Sangiovese wines (MS: Morellino di Scansano; TS: Toscana; CC: Chianti Classico; CR: Chianti Riserva) belonging to low (–L), medium (–M), and high (–H) price range, through the Multiple Factor Analysis ((**a**), groups representation; (**b**), individual factor map).

A great difference can be observed in the citation frequencies between trained assessors and consumers—on average 38% for all descriptors—indicating that trained assessors checked the astringency subqualities with more certainty than consumers, probably due to the previous training. The main descriptor used by consumers to define astringency of wines was dry (40% of the sample). Other terms were under 20%, and particularly, the least utilized was silk (7%). Similarly, the trained panel used the dry (76%) term most, but the least used to describe Sangiovese wines was velvet (41%). Hard was also highly used by trained assessors for Sangiovese; meanwhile, the frequency of citations by consumers ranged from 6% to 56% (mean 19%), indicating that this sensation was difficult to be perceived clearly. In order to underline differences between trained assessors and consumers in the evaluation of astringency subqualities of Sangiovese wines using CATA, a MFA was performed, as shown in Figure 2.

The first two dimensions of the MFA accounted for 57.96% of the variability of experimental data, representing 34.15% and 23.81% of the variance for the first and second dimension, respectively. In Figure 2a, the groups representation showed that trained assessors and consumers groups were adjacent, indicating that their sensory evaluations using the CATA terms slightly differed. The RV coefficients and *p*-values, calculated for the first and second dimension, were 0.676 (*p*-value = 0.025) and 0.715 (*p*-value = 0.025), respectively. The RV range 0.65–0.71 can represent an indicator of good agreement between configurations from consumers and trained panels [32].

In the individual factor map (Figure 2b), the wine spaces, compared through the partial range representation, were close except for Sangiovese wines CRH, CCH, and TSH. The different direction of projections means a high variability in defining these wines and indicates a strong disagreement between trained assessors and consumers for the high-level wines TSH and CRH. In fact, the consumers considered the TSH and CRH wines with negative sensations (dry, hard, aggressive) for 65% and 80%, in opposite to the trained assessors’ evaluation (50% for both wines), in which the silk, velvet, mouthcoat terms were selected for these wines (data available upon request).

### 3.2. Effect of Astringency Subqualities on Consumer Liking

**Figure 3:**
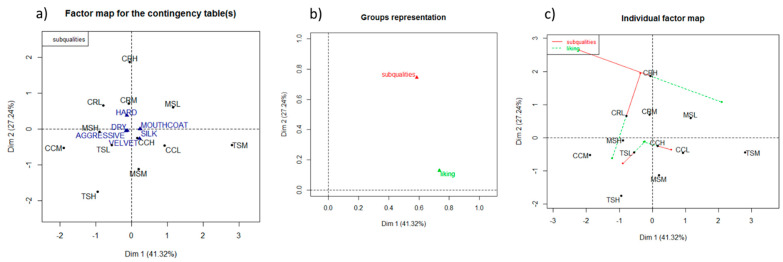
Preference map by the Multiple Factor Analysis (**a**), factor map for contingency table; (**b**), groups representation; (**c**), individual factor map on the consumers’ liking scores as a supplementary variable and CATA frequencies (from consumers) of astringency subqualities for Sangiovese wines (MS: Morellino di Scansano; TS: Toscana; CC: Chianti Classico; CR: Chianti Riserva) belonging to low (–L), medium (–M), and high (–H) price range.

In order to identify the drivers of liking for Sangiovese wine, the subqualities data corresponding to consumers and trained assessors were used to analyze the relationships with hedonic ratings from consumers in two separate preference maps. A MFA was performed on the citation frequencies by CATA and the liking scores by consumers. In Figure 3, the factor map for the contingency table (a), the groups representation (b), and the individual factor map (c) related to the six astringency terms and the twelve Sangiovese wines are shown.

**Figure 4:**
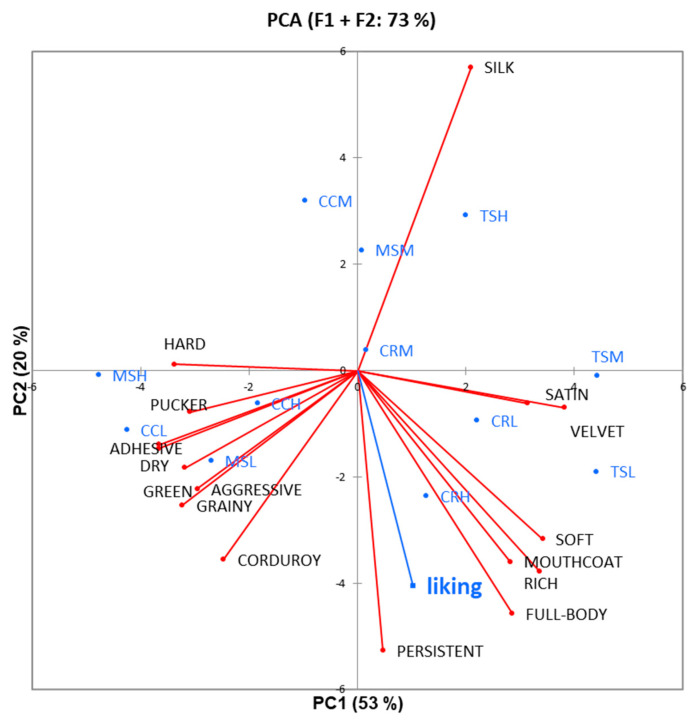
Multivariate data analysis (PCA) on the liking scores from consumers as a supplementary variable and intensity of astringency subqualities (RATA) by trained assessors for Sangiovese wines (MS: Morellino di Scansano; TS: Toscana; CC: Chianti Classico; CR: Chianti Riserva) belonging to low (–L), medium (–M), and high (–H) price range.

In Figure 4, the PCA showed the attributes of astringency obtained from trained assessors by RATA as active variables and the liking from consumers for the Sangiovese wines as the supplementary variable.

The first two dimensions explained the 73% of total variance. PCA displayed the separation of Sangiovese samples mainly on PC1, on which the attributes as hard (−0.802), pucker (−0.731), aggressive (−0.700), adhesive (−0.867), dry (−0.868), corduroy (−0.586), green (−0.754), and grainy (−0.766) were negatively projected, while satin (0.745), velvet (0.905), soft (0.811), mouthcoat (0.667), and rich (0.796) were positively projected. The liking (−0.590) was projected on the PC2 as the subqualities persistent (−0.768), rich (−0.551), full-body (−0.667) and mouthcoat (−0.524). These attributes related to a balanced astringency, full in the mouth, and rich in aroma, may represent the drivers of liking for wine consumers. This may indicate that together with mouthcoat, the other terms can apply in the CATA questionnaire to consumers. The term persistent, in particular, represents an overall sensation which comprises mouthfeel, taste, aroma and lasts over time, and can be suitable for red wine. On the other side, the term silk (0.833) had a negative effect on liking of Sangiovese wines, probably because this sensation was hardly recognized by consumers, as reported in Figure 1.

## 4. Discussion

In this study, a comparison in the evaluation of the astringency subqualities of Sangiovese wines by 150 regular wine consumers and trained assessors using a CATA questionnaire was made. The dry attribute was the most cited by both groups. This is not surprising because drying is the most common sensation used to describe astringency [14]. Hard, a term that involves bitterness and astringency, was also highly used by trained assessors for Sangiovese; meanwhile, for consumers, this sensation was difficult to be perceived. This may depend on their level of wine experience. For example, Lattey and colleagues [8] found a high discrepancy in bitterness perception between winemakers and consumers, which was considered negative for consumers and had a positive impact on winemakers’ evaluations. In the current study, the disagreement between consumers and trained assessors for astringency subqualities was related to the Toscana and Chianti Riserva wines belonging to the high price range (TSH and CRH). Indeed, while for trained assessors the presence of wood contributed to more positive sensations as silk, velvet, and mouthcoat, for consumers it was mainly associated with dry and bitter sensations.

Consumer panelists received no training and were only provided with definitions of the astringency subqualities, as opposed to the trained panellists who had received training and standards to demonstrate the different attributes during a long training period [16]. Consumers may have had some difficulties in recognising the sensations; in this sense, training may be more important than experience [33]. Fewer reports dealt with the training for astringency subqualities with touch standards [25,26], as well as the aforementioned coupled with CATA [16]. The training procedure indeed enhances practical tasting skills by increasing perceptual abilities and increases knowledge about product characteristics [34]—in our case, red wine. Therefore, the application of CATA questions with trained assessors has been recommended for the evaluation of complex products [23,35].

### Sangiovese Liking

A high discrepancy between consumers and trained assessors was found for wood-aged wines. Consumers liked Sangiovese wine as the Chianti Riserva high-price range, but considered the astringency as a negative feature, or they were not able to recognize the positive subqualities. Furthermore, the role of oak in winemaking is not well understood by most wine consumers, but knowledgeable consumers appreciate barrel-aged wines and are willing to pay a premium for them [36].

A Sangiovese wine, independently from denomination and price, is liked if it is characterised by mouthcoating tannin. In addition, characteristics as persistent, rich, and full-body represented liking drivers for consumers. Similarly, the soft, mouthcoat, and rich subqualities influenced the liking of commercial Tuscan Sangiovese wines positively [37]. Mouthcoat, velvet and complex were found to be the attributes able to describe the astringency of an ideal high-quality wine [14]. Some astringency subqualities such as round/smooth [38], or velvety [39] are regarded as more pleasing and contributed to astringency quality.

## 5. Conclusions

The evaluation of wine by consumers is paramount for wine producers in order to understand the drivers of preferences and attitudes. Mouthfeel sensations positively influence the quality of red wines but sometimes are difficult to discern. The negative astringency subqualities, in fact, were easier to be recognized with respect to the positive ones. Differences between consumers and trained assessors existed on the high price range wines, highlighting that the training with touch standard, even if time-consuming, is necessary when evaluating astringency subqualities of red wines. Consumers did not have full knowledge of astringency, mainly in relation to the positive subqualities. In particular, the effect of wood usage caused contrasting perceptions, being associated with negative subqualities by consumers, and positive by trained assessors. However, consumers liked the wood-aged wines. Mouthcoat was the term mainly associated with liking by consumers. In addition, other subqualities involving the overall flavor as persistent, rich, and full-body represented additional drivers of liking for Sangiovese wine.

This study has demonstrated that a trained sensory panel provides highly valuable information regarding the mouthfeel characteristics of Sangiovese wines and the attributes driving consumer liking. The study also offers insights to Sangiovese wine producers on which sensory characteristics positively (and negatively) influences liking. For instance, it underlines that a Sangiovese with mouthcoating tannins, rich in flavor, and full-bodied is of great interest for wine consumers.

## Figures and Tables

**Table 1:** Definitions of the sensory attributes presented in the Check-All-That-Apply (CATA) questionnaire to consumers and trained assessors. All the terms were presented to the trained assessors in Rate-All-That-Apply (RATA) questionnaire.

Attribute ^1^	Definition
*Dry*	*Feeling of luck of lubrication in mouth*
*Hard*	*Combined effect of astringency and bitterness*
*Aggressive*	*Excessive astringency of strong roughing nature*
*Silk*	*Tactile sensation like silk*
*Velvet*	*Tactile sensation like velvet*
*Mouthcoat*	*Like a coating film that adheres to mouth surfaces*
Rich	High flavor concentration with balanced astringency
Green	Combined effect of excess of acidity and astringency
Grainy	Sensation of micro-particles in mouth
Satin	A smooth and sliding astringency
Pucker	A reflex action of mouth surfaces being brought together and released in attempt to lubricate mouth surfaces
Full-Body	Sensation of high viscosity
Persistent	An overall sensation (flavor, tactile, taste) which lasts over time

^1^ Attributes and definitions in italics were presented in CATA questionnaire.

## Data Availability

The data presented in this study are not publicly available because ethical or privacy issues are present.
